# The Drought-Mediated Soybean GmNAC085 Functions as a Positive Regulator of Plant Response to Salinity

**DOI:** 10.3390/ijms22168986

**Published:** 2021-08-20

**Authors:** Xuan Lan Thi Hoang, Nguyen Nguyen Chuong, Tran Thi Khanh Hoa, Hieu Doan, Pham Hoang Phuong Van, Le Dang Minh Trang, Pham Ngoc Thai Huyen, Dung Tien Le, Lam-Son Phan Tran, Nguyen Phuong Thao

**Affiliations:** 1 Applied Biotechnology for Crop Development Research Unit, School of Biotechnology, International University, Quarter 6, Linh Trung Ward, Thu Duc City, Ho Chi Minh City 700000, Vietnam; htlxuan@hcmiu.edu.vn (X.L.T.H.); nguyenchuong1402@gmail.com (N.N.C.); ttkhoak11@gmail.com (T.T.K.H.); kaiba_doan@yahoo.com (H.D.); phphuongvan27@gmail.com (P.H.P.V.); minhtrangbt600@gmail.com (L.D.M.T.); huyenpham101697@gmail.com (P.N.T.H.); 2 Vietnam National University, Thu Duc City, Ho Chi Minh City 700000, Vietnam; 3 Agricultural Genetics Institute, Vietnam Academy of Agricultural Sciences, Pham Van Dong Str., Hanoi 100000, Vietnam; dung.le@bayer.com; 4 Department of Plant and Soil Science, Institute of Genomics for Crop Abiotic Stress Tolerance, Texas Tech University, Lubbock, TX 79409, USA

**Keywords:** *GmNAC085*, ROS-scavenging system, salt tolerance, stress-related genes, transcription factor

## Abstract

Abiotic stress factors, such as drought and salinity, are known to negatively affect plant growth and development. To cope with these adverse conditions, plants have utilized certain defense mechanisms involved in various aspects, including morphological, biochemical and molecular alterations. Particularly, a great deal of evidence for the biological importance of the plant-specific NAM, ATAF1/2, CUC2 (NAC) transcription factors (TFs) in plant adaptation to abiotic stress conditions has been reported. A previous *in planta* study conducted by our research group demonstrated that soybean (*Glycine max*) GmNAC085 mediated drought resistance in transgenic *Arabidopsis* plants. In this study, further characterization of GmNAC085 function in association with salt stress was performed. The findings revealed that under this condition, transgenic soybean plants overexpressing *GmNAC085* displayed better germination rates than wild-type plants. In addition, biochemical and transcriptional analyses showed that the transgenic plants acquired a better defense system against salinity-induced oxidative stress, with higher activities of antioxidant enzymes responsible for scavenging hydrogen peroxide or superoxide radicals. Higher transcript levels of several key stress-responsive genes involved in the proline biosynthetic pathway, sodium ion transporter and accumulation of dehydrins were also observed, indicating better osmoprotection and more efficient ion regulation capacity in the transgenic lines. Taken together, these findings and our previous report indicate that GmNAC085 may play a role as a positive regulator in plant adaptation to drought and salinity conditions.

## 1. Introduction

In recent years, soil salinization has emerged as one of the most serious abiotic stress factors, narrowing cultivable areas and threatening agricultural production [1]. According to a report in 2020, nearly 800 million hectares of land have been faced with saline problems [2]. Unfortunately, it is predicted that salinity will continue to spread to other parts of the world in the near future as a consequence of climate change and inappropriate farming practices [3]. It is known that under stress conditions, various biological processes in plants are negatively affected, including reduction in shoot growth, photosynthesis and biomass accumulation; promotion of senescence; and decrease in seed quantity and quality [4,5,6]. From an agricultural perspective, yield loss not only threatens global food security but also reduces the income of farmers.

A previous review on plant defense against salinity indicated that various synchronous mechanisms have been employed [7]. In response to external changes in general, salinity in particular, transcription factors (TFs) play important roles, as they can directly regulate gene expression [8]. Salinity-related TFs that have been identified are members of various TF families such as NAM, ATAF1/2, CUC2 (NAC), APETALA2/ethylene-responsive element-binding factor (AP2/EREBP), basic leucine zipper (bZIP), myeloblastosis (MYB) and WRKY [9,10]. Among these, the NAC TF family has been highlighted to perform pivotal functions in regulating different biological aspects of plant growth, development and plant responses to biotic and abiotic stresses [11,12,13,14]. In soybean (*Glycine max*), from the first large-scale examination of *NAC* gene expression, 9 out of 31 *GmNAC* genes in the soybean cv. Maverick displayed transcriptional induction upon various abiotic stress treatments, including dehydration, salinity and low temperature [15]. A subsequent investigation of the dehydration stress effect on expression of 152 full-length *GmNAC*s identified from the genome of soybean cv. Williams 82 (W82) revealed more dehydration-related *GmNAC* genes, of which 25 genes were upregulated and 6 genes were downregulated [16]. Following this study, a subset of these dehydration-responsive genes was selected for further expression profiling under drought stress conditions using two local soybean varieties (DT51 and MTD720) with contrasting drought-tolerant phenotypes [12,17]. Differential expression analyses of these genes helped the identification of certain members that might be associated with the drought tolerance capacity of soybean, including *GmNAC043*, *085*, *092*, *095*, *101* and *109*. A similar investigation carried out on drought-sensitive (B217 and H228) and drought-tolerant (Jindou74 and 78) soybean cultivars identified eight *GmNAC* genes with differential expression (*GmNAC004*, *021*, *065*, *066*, *073*, *082*, *083* and *087*) between the two studied groups of soybeans under drought stress conditions, whereby the tolerant cultivars displayed higher gene expression levels [18]. These findings indicate expression of drought-associated *GmNAC* genes is genotype-dependent.

Particularly, *GmNAC085* has been recognized as an important drought-related *NAC* gene, as its expression was the most induced by dehydration in both shoot and root tissues among the 25 *GmNAC* genes with upregulated expression in the study of Le et al. (2011) [16]. Expression of *GmNAC085* was also found to be significantly higher in the drought-tolerant cultivar than the drought-sensitive soybean cultivar under drought conditions [12,17]. Subsequent *in planta* functional characterization of *GmNAC085* highlighted the positive regulatory role of this TF in mediating plant response to drought. Transgenic *Arabidopsis* plants harboring *GmNAC085* acquired better drought tolerance with better reactive oxygen species (ROS) detoxification capacity owing to higher activities of the antioxidant enzymes superoxide dismutase (SOD), ascorbate peroxidase (APX), catalase (CAT), glutathione peroxidase (GPX), glutathione-S-transferase (GST) and glutathione reductase (GR) [19,20]. Furthermore, bioinformatic analyses revealed that the amino acid sequence of GmNAC085 protein was 39% identical to that of the rice (*Oryza sativa*) stress-responsive NAC1 (SNAC1/ONAC2) [16], which was found to improve the plant’s tolerance toward not only drought but also high salinity in different transgenic crop plants, including rice [21], wheat (*Triticum aestivum*) [22], cotton (*Gossypium hirsutum*) [23] and ramie (*Boehmeria nivea*) [24]. Similar to drought, salinity also triggers osmotic and oxidative stresses in plants [25,26]. Under these adverse environmental conditions, plants have difficulties in absorbing sufficient water due to decreased soil water potential, whereas excessive levels of endogenous ROS can trigger cellular damage and inhibition of metabolic activities [27]. Therefore, from the lines of evidence for the important role of GmNAC085 in mediating plant tolerance to drought, in this study, we further investigated the biological role of GmNAC085 in plant response to salinity to find out whether this TF is a plant regulator for multi-abiotic stress factors. To do this, effects of salinity on germination rate, antioxidant enzyme activities and expression of several key salinity-related genes were compared between transgenic soybean plants overexpressing *GmNAC085* and wild-type (WT) plants.

## 2. Results and Discussion

### 2.1. GmNAC085 Expression Is Inducible by Various Abiotic Stress Conditions

**Figure 1:**
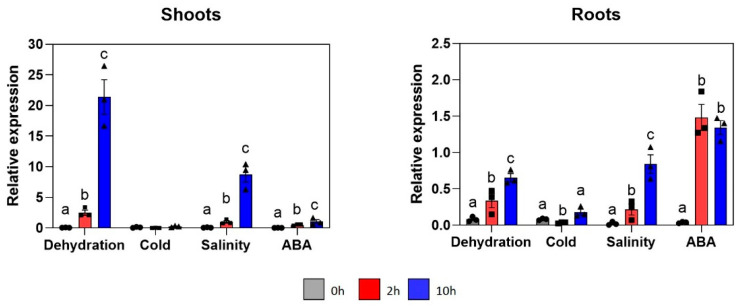
Expression profiles of *GmNAC085* in shoot and root tissues of soybean cultivar DT51 under dehydration, cold, salt and abscisic acid (ABA) treatments. Each value represents the mean ± SE (*n* = 3). Significance in transcriptional changes over the course of each treatment was analyzed by ANOVA and Tukey’s honestly significant difference and indicated by different letters (*p* < 0.05).

Environmental stress factors, such as drought, salinity and heat, seriously affect plant growth and development [28]. Under these adverse conditions, transcriptional regulation plays a crucial role in plant stress adaptation and tolerance [29]. Following our previous findings on the drought-responsive feature of GmNAC085 using local soybean DT51, which is a drought-tolerant cultivar [12,17], we further investigated the expression patterns of this TF-encoding gene in this cultivar that had been exposed to either dehydration, salinity, low temperature or abscisic acid (ABA). The obtained results showed that except cold treatment, *GmNAC085* expression was significantly upregulated over the course of dehydration, salinity and ABA challenge in both root and shoot tissues (Figure 1). These findings were in agreement with previous studies, as expression of several genes, including the *Arabidopsis Dehydration-responsive element* (*DRE*)-*binding factor 2* (*DREB2*) [30], *Galactinol synthase 1* (*AtGolS1*), *AtGolS2* [31] and the rice *OsNAC10* [32], was induced by drought and salinity but not by cold stress. Analysis of the *GmNAC085* regulatory region [12] also revealed that the promoter sequence did not contain (i) DRE *cis*-acting element, which has been known to involve in drought and cold response in an ABA-independent manner [33], or (ii) induction of C-repeat binding factor (CBF) expression regions (ICEr1/ICEr2), C-repeat (CRT) and low-temperature-responsive element (LTRE), which are cold-responsive *cis*-motifs [34]. In particular, transcript abundance of *GmNAC085* increased by 30-fold in the shoots and 5-fold in the roots after 2 h of dehydration, and 261-fold in the shoots and 8-fold in the roots after 10 h of dehydration (Figure 1). Upregulation of *GmNAC085* in the dehydration-treated W82 variety was also reported, with a higher induction level in the shoots than in the roots [16].

Under salinity conditions, transcript abundance of *GmNAC085* was enhanced by 115-fold and 50-fold in shoot and root tissues, respectively, after 10 h of treatment (Figure 1). *GmNAC085* expression was also ABA-inducible but at a higher level in the roots (16-fold) than in the shoots (9-fold) (Figure 1). Previously, the upregulation of the *Nine-cis-epoxycarotenoid dioxygenase 3* (*NCED3*) gene, whose product is a key enzyme in the ABA biosynthetic pathway, in *Arabidopsis* ectopically expressing *GmNAC085* was reported [19]. In addition, ABA-related *cis*-elements, including ABA-responsive element 2 (ABRE2) and the MYB recognition (MYBR) site, were also found within the promoter region of GmNAC085 [12,35], suggesting an interaction between ABA and GmNAC085 activities. Furthermore, it is noteworthy that dehydration and salinity induced *GmNAC085* more than ABA treatment in the shoot tissues, which was also observed in a study of transgenic Arabidopsis ectopically expressing the pearl millet (*Pennisetum glaucum*) *PgNAC21* [36]. A hypothesis proposed by Shinde et al. (2019) for this finding was a possible regulation of *GmNAC085* expression via an ABA-independent yet stress-dependent route [36]. Collectively, it is suggested that GmNAC085 might function in plant responses to various abiotic stress factors, and its role might vary in different tissues.

Importantly, amino acid sequence analysis revealed that GmNAC085 displayed 39% identity and 50% similarity to the well-known SNAC1 in rice, which functions as a positive regulator for plant response to drought [16]. In addition, both GmNAC085 and SNAC1 harbor sequences with transcriptional activation potential in the C-terminal region, as shown by the yeast one-hybrid assay [19,21], and are induced by dehydration, salinity and ABA treatments (Figure 1) [37]. Overexpression of *SNAC1* in rice resulted in enhanced tolerance toward drought and salinity [21,23]. GmNAC085, therefore, appears to be an excellent candidate to enhance salt stress tolerance of crop plants by genetic engineering.

### 2.2. GmNAC085-Transgenic Soybean Lines Display Normal Phenotype

**Figure 2:**
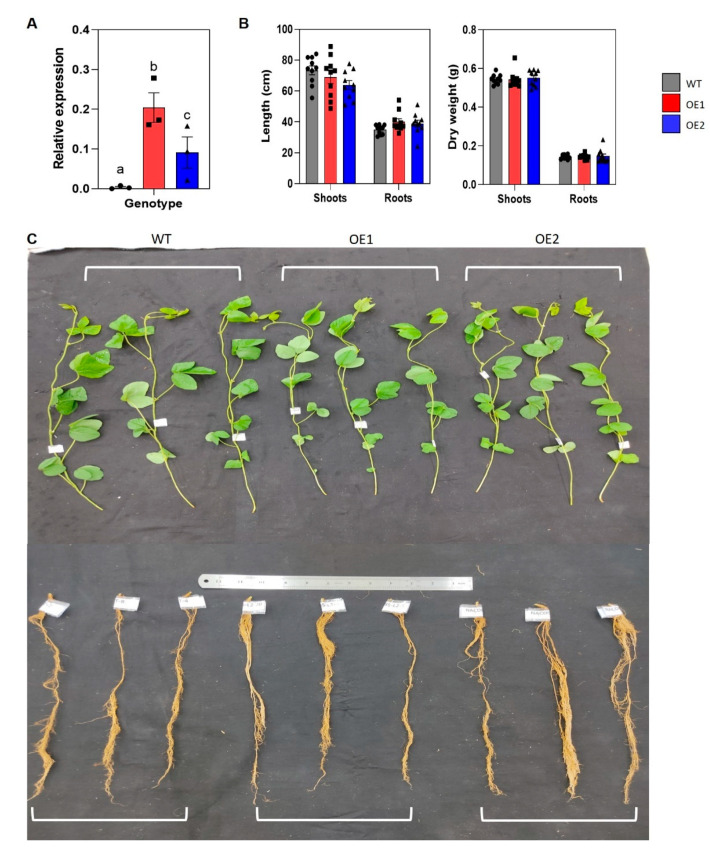
Expression of *GmNAC085* in 21-day-old *GmNAC085*-transgenic plants (OE1 and OE2) and their phenotype under normal growth conditions. (**A**) *GmNAC085* expression levels in wild-type (WT), OE1 and OE2 plants (*n* = 3). (**B**) Phenotypic parameters, including shoot length, root length, shoot dry weight and root dry weight (*n* = 10). (**C**) Representative pictures of shoots and roots of the WT and transgenic plants. Each value represents the mean ± SE. Significant differences analyzed by ANOVA and Tukey’s honestly significant difference test were indicated by different letters (*p* < 0.05).

To verify the biological role of GmNAC085 in relation to salt tolerance in soybean, we performed an *in planta* study using two independent homologous transgenic soybean lines overexpressing *GmNAC085* (OE1 and OE2). In comparison with WT plants, expression levels of *GmNAC085* in OE1 and OE2 were significantly higher, by 170-fold in OE1 and 58-fold in OE2 (Figure 2A). Analysis of shoot- and root-related traits, including shoot and root lengths and shoot and root dry weights, indicated that these two transgenic lines and WT showed similar plant growth and development under normal growth conditions (Figure 2B,C). A number of studies have reported growth retardation under normal conditions in transgenic plants using the promoter *35S* to drive expression of the transgene [38,39]. However, other overexpression studies reported no alteration in plant size due to activity of this constitutive promoter [40,41]. It has been suggested that although a smaller phenotype, including the transgenic *Arabidopsis* carrying *35S*::*GmNAC085*, is considered a non-desirable agronomic trait under non-stressed conditions, this could help the plants become more resilient to water deficit due to the lower demand of water consumption and better prevention in water loss [19,27,42,43]. Meanwhile, *35S*::*GmNAC085*-harboring transgenic soybean plants do not display this feature, as they share a similar morphology with WT plants under normal growth.

### 2.3. Transgenic Plants Have Higher Germination Rates under High Salinity Conditions

**Figure 3:**
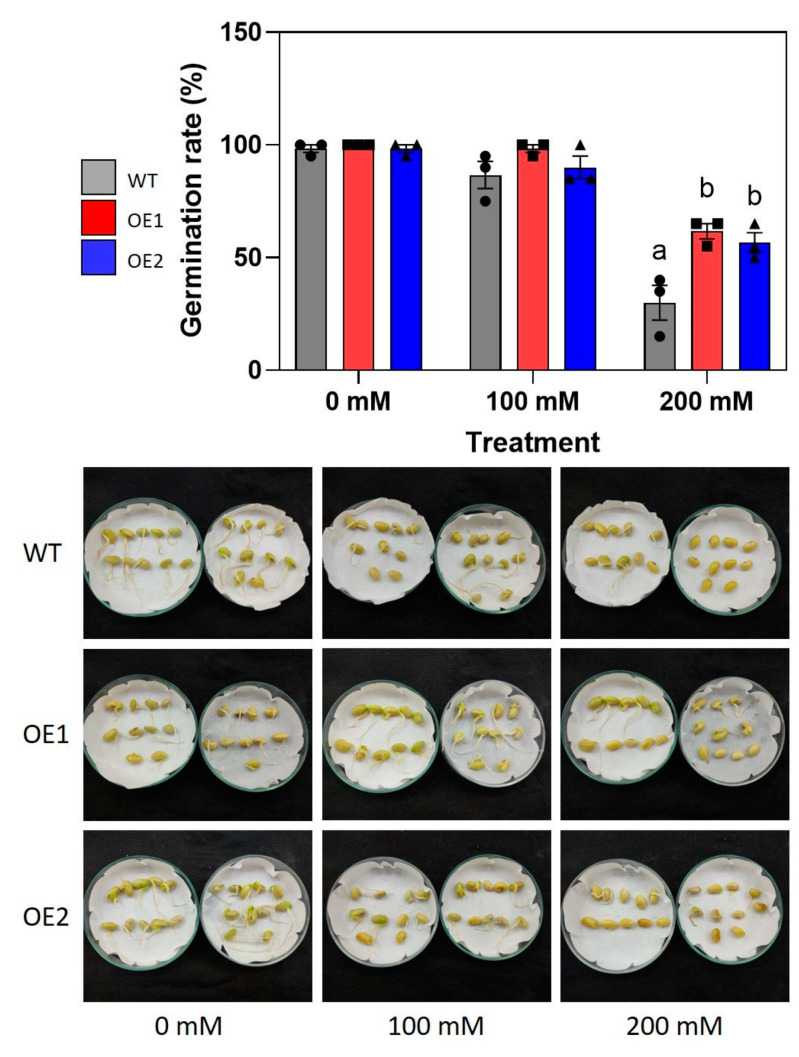
Germination of *GmNAC085*-transgenic (OE1 and OE2) and wild-type (WT) seeds under different concentrations of NaCl. The germination rates and representative pictures were taken after 3 days of incubating the seeds under dark conditions. Each value represents the mean ± SE (*n* = 3 replicates, 20 seeds/replicate). Significant differences among the genotypes in each treatment that were analyzed by ANOVA and Tukey’s honestly significant difference test were indicated by different letters (*p* < 0.05).

The importance of GmNAC085 in plant resistance to salinity was first evaluated using a germination assay. According to the obtained data, at a lower concentration of NaCl (100 mM), the germination rates of all three examined genotypes compared with their non-treated counterparts were similar (Figure 3). However, differential inhibitory effects of NaCl on soybean seed germination were clearly found under the high salt concentration of 200 mM. Under this stress condition, the germination rates of the soybean seeds were significantly reduced by 68% in WT, 38% in OE1 and 42% in OE2 compared with the control condition (Figure 3). This result indicated that *GmNAC085*-overexpressing plants maintained better germination rates than their non-transgenic counterparts under high salt concentrations. With influences on water uptake capacity and toxic ion disturbance on enzymatic activities, cellular metabolism and nutrient acquisition, salt stress is known to impair soybean seed germination and post-germinative growth, ultimately leading to yield loss [44,45]. Furthermore, NaCl treatment can cause oxidative stress, which is also detrimental to seed development and suppresses seed germination [46,47]. Therefore, the higher germination rates observed in the transgenic plants could be attributed to a better defensive capability against salt stress effects.

### 2.4. GmNAC085-Transgenic Plants Display Enhanced ROS-Scavenging Capacity

Prolonged salt stress triggers over-accumulation of ROS, leading to damage of cellular components, including DNA and proteins [48]. According to Sadak et al. (2020), salt stress significantly increases the hydrogen peroxide (H_2_O_2_) content in soybean leaves [49], which was also confirmed earlier in leaves of other plant species, such as sunflower (*Helianthus annuus*) [50] and wheat [51]. At appropriate concentrations, ROS can function as messenger molecules involved in acclimatory signaling to trigger plant tolerance against various abiotic stresses [52,53,54,55]. As ROS play dual roles in stress tolerance of plants, ROS synthesis and ROS-scavenging machineries are tightly regulated to maintain relevant levels of ROS at different plant developmental stages and under different growing environments [56]. In a previous study, transgenic *Arabidopsis* plants harboring *GmNAC085* were shown to obtain improved drought tolerance due to, at least partly, enhanced expression of genes associated with the activities of antioxidant enzymes, including SOD, CAT and APX that are known as the major ROS scavengers [19]. Therefore, expression profile analysis of antioxidant enzyme-encoding genes, *GmCAT*, *GmAPX1* and *GmMnSOD*, by quantitative real-time PCR (RT-qPCR) was carried out. 

**Figure 4:**
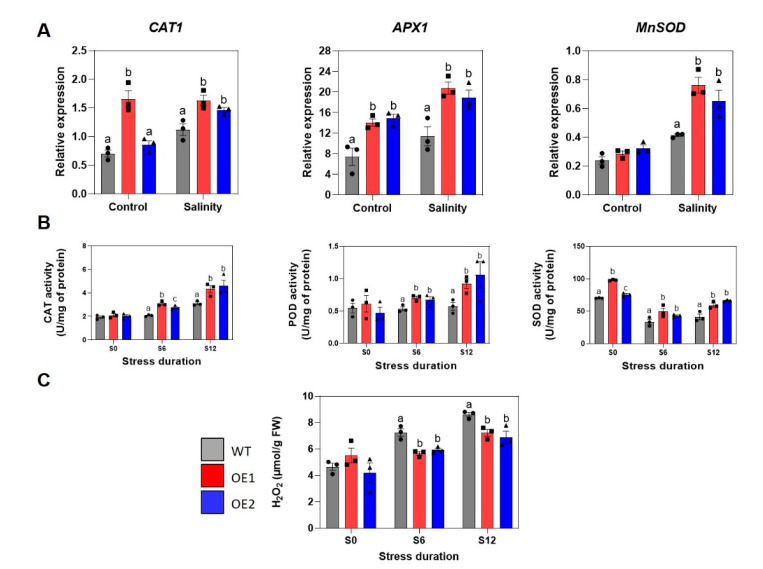
Evaluation of components of the reactive oxygen species-scavenging system in transgenic (OE1 and OE2) and wild-type (WT) plants under control and salinity conditions (S6 and S12 for 6-day and 12-day treatments, respectively). (**A**) Expression profile of antioxidant enzyme-encoding genes. (**B**) Enzymatic activities of catalase (CAT), peroxidase (POD) and superoxide dismutase (SOD). (**C**) Endogenous hydrogen peroxide (H_2_O_2_) content. Each value represents the mean ± SE (*n* = 3). Significant differences among the genotypes in each treatment that were analyzed by ANOVA and Tukey’s honestly significant difference test were indicated by different letters (*p* < 0.05).

As shown in Figure 4A, *GmNAC085* overexpression lines had increased transcript abundance of the examined antioxidant enzyme-encoding genes. Analyses of *GmCAT* expression patterns showed that only OE1 had significantly higher expression of this gene in non-stressed root tissue, probably due to higher *GmNAC085* transcript abundance compared with that of the OE2 line (Figure 2A). This high expression level status was maintained in the stressed OE1 line, whereas a substantial upregulation by 2.2-fold of *GmCAT* in the OE2 line upon salt treatment was observed (Figure 4A). Under normal conditions, expression levels of *GmAPX1* were higher in both transgenic lines compared with WT plants (by 2-fold in OE1 and 2.1-fold in OE2). After 10-day exposure to salinity, *GmNAC085*-overexpressing plants continuously outperformed their non-transgenic counterparts in *GmAPX1* transcript abundance. Following this, a significant increase in expression of *GmAPX1* was only observed in the transgenic lines, whereas the transcript level of this gene in the WT plants did not change much between the two conditions (Figure 4A). Regarding *GmMnSOD*, the transcript abundance of this gene was found almost identical among the three genotypes under normal conditions but at substantially higher levels under salt treatment in the transgenic lines (by 2.6-fold in OE1 and 1.6-fold in OE2 in comparison with the WT counterpart) (Figure 4A).

Data from biochemical assays were also in agreement with the RT-qPCR analyses. Activities of CAT and peroxidase (POD), which are H_2_O_2_-scavenging enzymes, remained relatively low and similar among the three studied genotypes under normal conditions (Figure 4B). Under the applied stress condition, though all the three genotypes enhanced activities of these enzymes, the overexpression lines displayed their activities at remarkably higher levels. This partially explains the lower H_2_O_2_ accumulation after 6-day and 12-day stress application in the transgenic plants than in WT plants (Figure 4C). 

Interestingly, activities of SOD, which is responsible for the dismutation of superoxide into H_2_O_2_, were found to be more active in the transgenic plants under both non-stressed and stressed conditions (at least by 1.4-fold and 1.3-fold higher in OE1 and OE2, respectively). It is also noted that under the stress conditions, molecular analyses revealed upregulation of *GmMnSOD*, whereas the biochemical data did not demonstrate this trend (Figure 4A,B). This could be due to alteration in expression of other genes encoding GmSOD isozymes, which remains to be explored. In addition, according to our data, it can be deduced that the increase in H_2_O_2_ over the course of stress treatment in general could be due to the over-production of this ROS from various sources that employ different enzymes, such as photorespiration, electron transport chain and redox reactions in the apoplast [57,58], rather than depending on the superoxide conversion into H_2_O_2_ by SOD enzyme activity (Figure 4B,C).

Many studies have shown that overexpression of *CAT* can increase plant resistance to abiotic and biotic stresses [59,60], acknowledging its indispensable role in alleviating oxidative stress [61,62,63]. With APX, this is a group of enzymes that belongs to the POD superfamily and plays a central role in the ascorbate-glutathione cycle that has evolved in plants to scavenge H_2_O_2_ from plant chloroplasts and cytosol [64]. Regarding *SOD* genes, they can be divided into four subfamilies, among which three (*MnSOD*, *FeSOD* and *Cu/ZnSOD*) are widely found in plants and one (*NiSOD*) is present in streptomyces [65,66]. Frequently, members of different SOD subfamilies are localized to different cellular compartments, including mitochondria (MnSOD), peroxisomes (MnSOD and Cu/ZnSOD), chloroplasts and cytosol (FeSOD, Cu/ZnSOD) [67]. The important role of the *APX* and *SOD* gene families in antioxidative stress has now been demonstrated in a variety of plants. For example, transgenic cassava (*Manihot esculenta*) co-expressing cytoplasmic *MeCu/ZnSOD* and *MeAPX2* displayed high levels of SOD and APX antioxidant enzyme activities, thus improving their tolerance to cold stress [68]. In another report, ectopic expression of *MnSOD* gene from *Tamarix androssowii* conferred salinity and oxidative stress tolerance in the transgenic poplar (*Populus davidiana* x *P. bolleana*) [69]. Furthermore, various studies have reported that the enhanced stress tolerance in plants harboring a regulatory transgene (e.g., *NAC* and *MYB*) such as *GmNAC085*-transgenic *Arabidopsis* [19], *GmNAC20*-transgenic rice [70] and *SlMYB102*-transgenic tomato (*Solanum lycopersicum*) [71] or a non-antioxidant functional transgene (e.g., *Sodium–proton antiporter* (*NHX*) and *Salt overly sensitive* (*SOS*)) such as *TaNHX2*-transgenic sunflower [72], *AtNHX1*-transgenic mung bean (*Vigna radiata*) [73] and *GmsSOS1*-transgenic *Arabidopsis* [74] was also associated with higher expression levels of antioxidant enzyme-encoding genes and/or higher activities of antioxidant enzymes.

Taken these lines of evidence together, the higher activities of CAT, POD and SOD and higher transcript abundance of antioxidant enzyme-encoding genes in transgenic plants indicated their better defense capability against oxidative stress compared with the non-transgenic counterpart. As oxidative stress was found to be associated with seed germination [46], these results may also explain the higher germination rates observed in the transgenic plants compared with WT plants under NaCl treatment (Figure 3 and Figure 4). 

### 2.5. Expression Levels of Other Stress-Related Genes Are Also Enhanced in Transgenic Plants under Salinity Conditions

**Figure 5:**
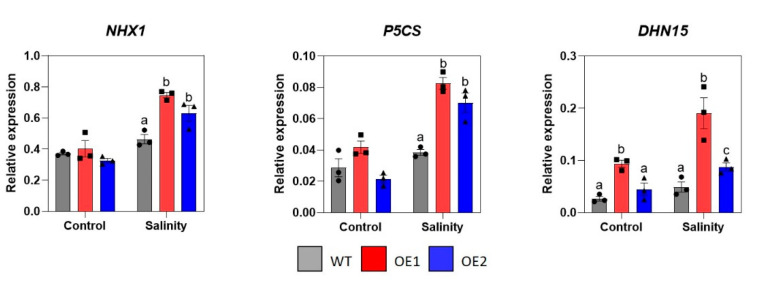
Expression profiles of three stress-related genes in root tissues of *GmNAC085*-transgenic soybean lines (OE1 and OE2) and wild-type (WT) plants under salt stress treatments, including *GmNHX1*, *GmP5CS* and *GmDHN15*. Each value represents the mean ± SE (*n* = 3). Significant differences among the genotypes in each treatment that were analyzed by ANOVA and Tukey’s honestly significant difference test were indicated by different letters (*p* < 0.05).

In addition to antioxidant genes, expression of the Na^+^/H^+^ antiporter-encoding gene (*GmNHX1*), as well as two osmoprotectant-related genes *Delta-1-pyrroline-5-carboxylase synthase* (*GmP5CS*) and *Dehydrin 15* (*GmDHN15*), was also analyzed. Generally, the expression of these genes was upregulated upon stress exposure, which is consistent with findings from previous studies [75,76,77]. However, both transgenic lines displayed higher induction levels than WT plants (Figure 5). It is noticed that the *GmNHX1* and *GmP5CS* shared similar expression patterns to that of *GmMnSOD* (Figure 4A and Figure 5). In particular, transcript abundance of these genes was at the same level among the three examined genotypes under normal conditions but remarkably higher in the overexpression lines upon stress application (at least 1.5-fold higher for *NHX1* and 1.8-fold higher for *P5CS*). Meanwhile, *DHN15* had the highest expression activity in the OE1 in both non-stressed (3.6-fold higher compared to WT) and stressed conditions (3.9-fold higher compared to WT) (Figure 5). 

Salinity, along with drought, cause changes in osmotic pressure and oxidative stress that trigger cellular damage and dehydration [78]. To deal with this, several strategies can be used by plants, including accumulation of osmolytes to lower cellular osmotic potential, activation of antioxidant systems for ROS removal and increase in chaperone activities for protein protection [79]. For example, synthesis of proline, an amino acid that can function as an osmolyte and osmoprotectant, is usually promoted under the stress condition by enhancement activities of P5CS, the rate-limiting enzyme in the proline biosynthetic pathway [75,80]. Similarly, increased synthesis of dehydrin and late embryogenesis abundant (LEA) proteins that play important roles in protein protection is also observed [77,81]. In this study, the upregulated expression of *P5CS* and *DHN15* would bring certain advantages for the *GmNAC085*-transgenic soybean lines in mitigating the salinity effects. It is also known that the reluctant accumulation of cellular Na^+^ under salinity conditions leads to the disruption of ion balance and cellular metabolism [82,83]. Compartmentalization of Na^+^ ions in vacuole by activity of vacuolar Na^+^/H^+^ antiporter in replacement of Na^+^ for H^+^ has long been proposed as an effective mechanism for salt tolerance [84]. This helps avoid deleterious effects of excessive Na^+^ in the cytosol, while the osmotic balance in the vacuole can be maintained by using Na^+^ as an ionic osmolyte [85,86,87]. Furthermore, it has been evidenced that overexpression of *Arabidopsis* vacuolar *NHX1* could confer improved salt tolerance in transgenic tomato plants [88]. Similar findings were also reported for other transgenic crop species overexpressing *NHX*-encoding genes, including rice [89,90,91], wheat [92], barley (*Hordeum vulgare*) [93], cowpea (*Vigna unguiculata*) [94] and mung bean [73]. Therefore, enhanced transcriptional activities of *GmNHX1* observed in the *GmNAC085*-transgenic plants would contribute to the maintenance of normal metabolism under salt stress. Additionally, transcriptional activation assay in yeast demonstrated that the C-terminal transcriptional regulatory region of GmNAC085 possesses a transcriptional activation domain that enables the protein to function as a transcriptional activator [19]. Therefore, higher expression levels of the examined genes observed in this study could be the results of direct and/or indirect regulation of this NAC TF. 

## 3. Materials and Methods

### 3.1. Plant Materials and Plant Growth Conditions

Seeds of soybean varieties W82 and DT51 were obtained from RIKEN Center (Yokohama, Japan) and Legumes Research and Development Center (Hanoi, Vietnam), respectively. The transgenic soybean (W82 background) lines were generated by the service at Iowa State University (Ames, IA, USA) using the *Agrobacterium tumefaciens*-mediated transformation method. Cassette *P_35S_-GmNAC085*-*NOS* from a pGKX vector constructed previously [19,95] was cloned into pENTR Direction TOPO using the following primers: 5′-CACCGAGCTTGCCAACATGGTGGAG-3′ (forward) and 5′-CGATCTAGTAACATAGATGAC-3′ (reverse). Subsequently, the cassette was transferred into a pTF101.1gw1 vector and then used for transformation. The homozygous transgenic progenies used in this study were verified as independent lines according to Mendelian segregation analyses for the ratio of Basta-resistant/Basta-sensitive phenotypes, followed by molecular confirmation [96,97]. The plants were grown in plastic pots containing soil, coir, husk ash and compost (Tribat soil, Saigon Xanh Biotechnology Ltd. Company, Ho Chi Minh City, Vietnam) and under net house conditions (28–33 °C, 60–70% humidity and natural photoperiod) [12].

### 3.2. Abiotic Stress Assays for Analyses of GmNAC085 Expression

Local soybean variety DT51 was used for various stress challenges, as described by Tran et al. (2009) [15]. For dehydration treatment, 12-day-old plants were carefully pulled from the container and washed to remove soil attached to the root surfaces. The plants were then placed on filter papers and allowed to dehydrate in a controlled growth chamber (60% relative humidity, 28 °C day/night temperature and 200 µmol m^–2^ s^–1^ light intensity). For salinity and ABA treatments, plants were transferred to 250 mM NaCl and 100 µM ABA solution, respectively, under laboratory conditions. Low-temperature treatment was conducted by keeping the seedlings in distilled water maintained at 4 °C. During the assays, the roots and shoots tissues of treated plants were collected at 0, 2- and 10-h timepoints for expression analysis of *GmNAC085*. All experiments were carried out with three biological replicates.

### 3.3. Morphological Analysis of Transgenic Plants under Normal Conditions

Root and shoot growth of V4-stage seedlings (i.e., 21-day-old) were evaluated for length and dry biomass. The seedlings were grown in plastic pots (10 cm in diameter and 80 cm in height, one plant per pot) with normal irrigation until they were harvested for the measurement (*n* = 10) [98]. 

### 3.4. Analysis of Seed Germination Rate

For this experiment, seeds were first sterilized using 2% sodium hypochlorite (NaOCl) for 10 min before they were rinsed with distilled water for chemical removal. Next, the seeds were incubated between two layers of filter paper placed in a Petri dish (9 cm in diameter) supplied with 10 mL of NaCl solution with different concentrations (0, 100 and 200 mM) [99,100]. After keeping the plates in dark conditions at room temperature for three days, the germination rates were recorded. Seeds were considered successfully germinated if the length of rising radicles was at least greater than half of the seed length [101]. For each genotype, three replications per sodium chloride concentration were used, of which each replication was one plate with 20 seeds. 

### 3.5. Biochemical Analyses for Endogenous Hydrogen Peroxide Content and Antioxidant Enzyme Activities

To initiate salt stress, 12-day healthy seedlings of both transgenic and non-transgenic plants were irrigated with NaCl solution (100 mM) (100 mL/plant) every two days. For each genotype, the leaf tissues of three individual plants (*n* = 3, 0.2 g/replicate) were collected on days 0, 6th and 12th during the stress application. Previously described methods for determination of contents of H_2_O_2_ [43,102] and soluble proteins [103], as well as activities of CAT [104], SOD [105] and POD [106] enzymes, were used.

### 3.6. Gene Expression Analysis by RT-qPCR

Expression analysis of *GmNAC085* in DT51 root and shoot tissues that were exposed to various abiotic stress conditions (Section 3.2) and expression analysis of stress-related genes in root tissues of transgenic and WT plants subjected to salinity (Section 3.5) for ten days were conducted using RT-qPCR. The primer sets used for this assay are provided in Table 1. Total RNA extraction and purification, cDNA synthesis and RT-qPCR were carried out using commercial kits (Thermo Scientific, Waltham, MA, USA) and following the guidelines provided by the manufacturer [97]. NanoDrop One^C^ Microvolume UV-Vis Spectrophotometer (ND-ONEC-W, Thermo Scientific, MA, USA) was used to determine the concentrations and quality of total RNA extracts. cDNA synthesis was carried out using the same amount of total RNA from each sample. Preparation for reactions, thermal profile and melting curve analysis in RT-qPCR assay (Mastercycler® ep *realplex*, Eppendorf, Hamburg, Germany) was described in our previous study [97]. *Fbox* [107] was used as the reference gene for normalization based on the 2^−Δ*Ct*^ method [108]. LinRegPCR software (version 2020.2, Academic Medical Center, Amsterdam, The Netherlands) was used to calculate the efficiency of PCR reactions. 

**Table 1:** Information of primers that were used in gene expression analysis.

Genes	ID	Primer Type	Primer Sequence (5′-3′)	Amplicon Size (bp)	References
*GmNAC085*	*Glyma12g22880*	Forward	GGCTAGACACATACAATGAATCGG	92	[16]
		Reverse	TGCGGTGCTGTGGTGAAA		
*GmFbox*	*Glyma12g051100*	Forward	AGATAGGGAAATTGTGCAGGT	93	[107]
		Reverse	CTAATGGCAATTGCAGCTCTC		
*GmCAT*	*Glyma06g017900*	Forward	CCACAGCCATGCCACTCAAG	184	[109]
		Reverse	CAGGACCAAGCGACCAACAG		
*GmAPX1*	*Glyma12g073100*	Forward	AGTTGGCTGGCGTTGTTG	86	[109]
		Reverse	TGGTGGCTCAGGCTTGTC		
*GmMnSOD*	*Glyma04g221300*	Forward	GCACCACCAGACTTACATCAC	88	[109]
		Reverse	AACGACGGCGGAGGAATC		
*GmNHX1*	*Glyma20g229900*	Forward	CTTTCCACTCCAACACACAC	110	[76]
		Reverse	GGTGAGCCAGGTTCTATAGG		
*GmP5CS*	*Glyma18g034300*	Forward	TGTCTCTCAGATCAAGAGTTCCAC	144	[110]
		Reverse	CAGCCTGCTGGATAGTCTATTTTT		
*GmDHN15*	*Glyma11g149900*	Forward	TTTTGTTTTGTTGTATTGTGTAG	150	[77]
		Reverse	GAAAAATCCTCCACCTGACGA		

### 3.7. Statistical Analyses

Data were analyzed using one-way ANOVA and Tukey’s honestly significant difference test for comparison among the examined genotypes under the same treatment to identify statistically significant differences (*p* < 0.05).

## 4. Conclusions

The results from this study showed that GmNAC085 functions as a positive regulator for plant response to salinity, in addition to a previous report on its contribution to plant resistance to drought. Under high salinity conditions, *GmNAC085*-overexpressing soybean plants maintained better germination rates and had more robust antioxidant enzyme activities. Gene expression profiling data also indicate that the enhanced salinity tolerance mediated by GmNAC085 comes from the increased biosynthesis of osmoprotectants proline and dehydrin, as well as effective sequestration of excessive cytosolic Na^+^ using the vacuolar Na^+^/H^+^ antiporter. Therefore, the findings presented here, together with our previous report, should lay a solid foundation for further study into the molecular mechanisms by which GmNAC085 mediates multi-responses to different types of osmotic stress, as well as for the development of stress-tolerant crops based on GmNAC085 manipulation.

## Data Availability

The data presented in this study are available on request from the corresponding author.

## References

[1] Qados A.M.S.A. (2011). Effect of Salt Stress on Plant Growth and Metabolism of Bean Plant *Vicia faba* (L.). J. Saudi Soc. Agric. Sci..

[2] Krishnamurthy S.L., Pundir P., Warraich A.S., Rathor S., Lokeshkumar B.M., Singh N.K., Sharma P.C. (2020). Introgressed Saltol QTL Lines Improves the Salinity Tolerance in Rice at Seedling Stage. Front. Plant Sci..

[3] Bekmirzaev G., Ouddane B., Beltrao J., Fujii Y. (2020). The Impact of Salt Concentration on the Mineral Nutrition of *Tetragonia tetragonioides*. Agriculture.

[4] Manchanda G., Garg N. (2008). Salinity and Its Effects on the Functional Biology of Legumes. Acta Physiol. Plant..

[5] Al-shareef N.O., Tester M. (2019). Plant Salinity Tolerance. eLS.

[6] Li M., Chen R., Jiang Q., Sun X., Zhang H., Hu Z. (2021). GmNAC06, a NAC Domain Transcription Factor Enhances Salt Stress Tolerance in Soybean. Plant Mol. Biol..

[7] Rejeb I.B., Pastor V., Mauch-Mani B. (2014). Plant Responses to Simultaneous Biotic and Abiotic Stress: Molecular Mechanisms. Plants.

[8] Khan S.-A., Li M.-Z., Wang S.-M., Yin H.-J. (2018). Revisiting the Role of Plant Transcription Factors in the Battle against Abiotic Stress. Int. J. Mol. Sci..

[9] Hoang X.L.T., Nhi D.N.H., Thu N.B.A., Thao N.P., Tran L.-S.P. (2017). Transcription Factors and Their Roles in Signal Transduction in Plants under Abiotic Stresses. Curr. Genomics.

[10] Zhao C., Zhang H., Song C., Zhu J.-K., Shabala S. (2020). Mechanisms of Plant Responses and Adaptation to Soil Salinity. Innov..

[11] Tran L.-S.P., Nakashima K., Sakuma Y., Simpson S.D., Fujita Y., Maruyama K., Fujita M., Seki M., Shinozaki K., Yamaguchi-Shinozaki K. (2004). Isolation and Functional Analysis of *Arabidopsis* Stress-Inducible NAC Transcription Factors That Bind to a Drought-Responsive *cis*-Element in the *Early Responsive to Dehydration Stress 1* Promoter. Plant Cell.

[12] Thao N.P., Thu N.B.A., Hoang X.L.T., van Ha C., Tran L.S.P. (2013). Differential Expression Analysis of a Subset of Drought-Responsive *GmNAC* Genes in Two Soybean Cultivars Differing in Drought Tolerance. Int. J. Mol. Sci..

[13] Erpen L., Devi H.S., Grosser J.W., Dutt M. (2018). Potential Use of the DREB/ERF, MYB, NAC and WRKY Transcription Factors to Improve Abiotic and Biotic Stress in Transgenic Plants. Plant Cell Tissue Organ Cult..

[14] Sun H., Hu M., Li J., Chen L., Li M., Zhang S., Zhang X., Yang X. (2018). Comprehensive Analysis of NAC Transcription Factors Uncovers Their Roles during Fiber Development and Stress Response in Cotton. BMC Plant Biol..

[15] Tran L.-S.P., Quach T.N., Guttikonda S.K., Aldrich D.L., Kumar R., Neelakandan A., Valliyodan B., Nguyen H.T. (2009). Molecular Characterization of Stress-Inducible *GmNAC* Genes in Soybean. Mol. Genet. Genomics.

[16] Le D.T., Nishiyama R.I.E., Watanabe Y., Mochida K., Yamaguchi-Shinozaki K., Shinozaki K., Tran L.-S.P. (2011). Genome-Wide Survey and Expression Analysis of the Plant-Specific NAC Transcription Factor Family in Soybean during Development and Dehydration Stress. DNA Res..

[17] Thu N.B.A., Hoang X.L.T., Doan H., Nguyen T.-H., Bui D., Thao N.P., Tran L.-S.P. (2014). Differential Expression Analysis of a Subset of *GmNAC* Genes in Shoots of Two Contrasting Drought-Responsive Soybean Cultivars DT51 and MTD720 under Normal and Drought Conditions. Mol. Biol. Rep..

[18] Hussain R.M., Ali M., Feng X., Li X. (2017). The Essence of *NAC* Gene Family to the Cultivation of Drought-Resistant Soybean (*Glycine max* L. Merr.) Cultivars. BMC Plant Biol..

[19] Nguyen K.H., Mostofa M.G., Li W., van Ha C., Watanabe Y., Le D.T., Thao N.P., Tran L.S.P. (2018). The Soybean Transcription Factor GmNAC085 Enhances Drought Tolerance in *Arabidopsis*. Environ. Exp. Bot..

[20] Nguyen K.H., Mostofa M.G., Watanabe Y., Tran C.D., Rahman M.M., Tran L.-S.P. (2019). Overexpression of *GmNAC085* Enhances Drought Tolerance in *Arabidopsis* by Regulating Glutathione Biosynthesis, Redox Balance and Glutathione-Dependent Detoxification of Reactive Oxygen Species and Methylglyoxal. Environ. Exp. Bot..

[21] Hu H., Dai M., Yao J., Xiao B., Li X., Zhang Q., Xiong L. (2006). Overexpressing a NAM, ATAF, and CUC (NAC) Transcription Factor Enhances Drought Resistance and Salt Tolerance in Rice. Proc. Natl. Acad. Sci. USA.

[22] Saad A.S.I., Li X., Li H.-P., Huang T., Gao C.-S., Guo M.-W., Cheng W., Zhao G.-Y., Liao Y.-C. (2013). A Rice Stress-Responsive *NAC* Gene Enhances Tolerance of Transgenic Wheat to Drought and Salt Stresses. Plant Sci..

[23] Liu G., Li X., Jin S., Liu X., Zhu L., Nie Y., Zhang X. (2014). Overexpression of Rice NAC Gene *SNAC1* Improves Drought and Salt Tolerance by Enhancing Root Development and Reducing Transpiration Rate in Transgenic Cotton. PLoS ONE.

[24] An X., Liao Y., Zhang J., Dai L., Zhang N., Wang B., Liu L., Peng D. (2015). Overexpression of Rice NAC Gene *SNAC1* in Ramie Improves Drought and Salt Tolerance. Plant Growth Reg..

[25] Uddin M.N., Hossain M.A., Burritt D.J., Ahmad P. (2016). Salinity and drought stress: Similarities and differences in oxidative responses and cellular redox regulation. Water Stress and Crop Plants: A Sustainable Approach.

[26] Pinheiro D.T., Delazari F., Nick C., Mattiello E.M., dos Santos Dias D.C.F. (2019). Emergence and Vegetative Development of Melon in Function of the Soil Salinity. Aust. J. Crop Sci..

[27] Hoang X.L.T., Nguyen N.C., Nguyen Y.N.H., Watanabe Y., Tran L.S.P., Thao N.P. (2020). The Soybean GmNAC019 Transcription Factor Mediates Drought Tolerance in *Arabidopsis* in an Abscisic Acid-Dependent Manner. Int. J. Mol. Sci..

[28] Wang C.-T., Ru J.-N., Liu Y.-W., Li M., Zhao D., Yang J.-F., Fu J.-D., Xu Z.-S. (2018). Maize WRKY Transcription Factor ZmWRKY106 Confers Drought and Heat Tolerance in Transgenic Plants. Int. J. Mol. Sci..

[29] Wang H., Meng J., Peng X., Tang X., Zhou P., Xiang J., Deng X. (2015). Rice WRKY4 Acts as a Transcriptional Activator Mediating Defense Responses toward *Rhizoctonia solani*, the Causing Agent of Rice Sheath Blight. Plant Mol. Biol..

[30] Liu Q., Kasuga M., Sakuma Y., Abe H., Miura S., Yamaguchi-Shinozaki K., Shinozaki K. (1998). Two Transcription Factors, DREB1 and DREB2, with an EREBP/AP2 DNA Binding Domain Separate Two Cellular Signal Transduction Pathways in Drought-and Low-Temperature-Responsive Gene Expression, Respectively, in *Arabidopsis*. Plant Cell.

[31] Taji T., Ohsumi C., Iuchi S., Seki M., Kasuga M., Kobayashi M., Yamaguchi-Shinozaki K., Shinozaki K. (2002). Important Roles of Drought-and Cold-inducible Genes for Galactinol Synthase in Stress Tolerance in *Arabidopsis thaliana*. Plant J..

[32] Jeong J.S., Kim Y.S., Baek K.H., Jung H., Ha S.-H., do Choi Y., Kim M., Reuzeau C., Kim J.-K. (2010). Root-Specific Expression of *OsNAC10* Improves Drought Tolerance and Grain Yield in Rice under Field Drought Conditions. Plant Physiol..

[33] Shinozaki K., Yamaguchi-Shinozaki K. (2000). Molecular Responses to Dehydration and Low Temperature: Differences and Cross-Talk between Two Stress Signaling Pathways. Curr. Opin. Plant Biol..

[34] Yamaguchi-Shinozaki K., Shinozaki K. (2005). Organization of *cis*-Acting Regulatory Elements in Osmotic- and Cold-Stress-Responsive Promoters. Trends Plant Sci..

[35] Shukla P.S., Agarwal P., Gupta K., Agarwal P.K. (2015). Molecular Characterization of an MYB Transcription Factor from a Succulent Halophyte Involved in Stress Tolerance. AoB Plants.

[36] Shinde H., Dudhate A., Tsugama D., Gupta S.K., Liu S., Takano T. (2019). Pearl Millet Stress-Responsive NAC Transcription Factor PgNAC21 Enhances Salinity Stress Tolerance in *Arabidopsis*. Plant Physiol. Biochem..

[37] Nuruzzaman M., Manimekalai R., Sharoni A.M., Satoh K., Kondoh H., Ooka H., Kikuchi S. (2010). Genome-Wide Analysis of NAC Transcription Factor Family in Rice. Gene.

[38] Liao X., Guo X., Wang Q., Wang Y., Zhao D., Yao L., Wang S., Liu G., Li T. (2017). Overexpression of *MsDREB6.2* Results in Cytokinin-deficient Developmental Phenotypes and Enhances Drought Tolerance in Transgenic Apple Plants. Plant J..

[39] Du X., Li W., Sheng L., Deng Y., Wang Y., Zhang W., Yu K., Jiang J., Fang W., Guan Z. (2018). Over-Expression of *Chrysanthemum CmDREB6* Enhanced Tolerance of *Chrysanthemum* to Heat Stress. BMC Plant Biol..

[40] Guan Q., Liao X., He M., Li X., Wang Z., Ma H., Yu S., Liu S. (2017). Tolerance Analysis of Chloroplast *OsCu/Zn-SOD* Overexpressing Rice under NaCl and NaHCO_3_ Stress. PLoS ONE.

[41] Yin X., Cui Y., Wang M., Xia X. (2017). Overexpression of a Novel MYB-Related Transcription Factor, *OsMYBR1*, Confers Improved Drought Tolerance and Decreased ABA Sensitivity in Rice. Biochem. Biophys. Res. Commun..

[42] Kong X., Zhou S., Yin S., Zhao Z., Han Y., Wang W. (2016). Stress-Inducible Expression of an F-Box Gene *TaFBA1* from Wheat Enhanced the Drought Tolerance in Transgenic Tobacco Plants without Impacting Growth and Development. Front. Plant Sci..

[43] Nguyen N.C., Hoang X.L.T., Nguyen Q.T., Binh N.X., Watanabe Y., Thao N.P., Tran L.S.P. (2019). Ectopic Expression of *Glycine max GmNAC109* Enhances Drought Tolerance and ABA Sensitivity in *Arabidopsis*. Biomolecules.

[44] Hamayun M., Hussain A., Khan S.A., Irshad M., Khan A.L., Waqas M., Shahzad R., Iqbal A., Ullah N., Rehman G. (2015). Kinetin Modulates Physio-Hormonal Attributes and Isoflavone Contents of Soybean Grown under Salinity Stress. Front. Plant Sci..

[45] Rajabi Dehnavi A., Zahedi M., Ludwiczak A., Cardenas Perez S., Piernik A. (2020). Effect of Salinity on Seed Germination and Seedling Development of Sorghum (*Sorghum Bicolor* (L.) Moench) Genotypes. Agronomy.

[46] Ma Q., Kang J., Long R., Zhang T., Xiong J., Zhang K., Wang T., Yang Q., Sun Y. (2017). Comparative Proteomic Analysis of Alfalfa Revealed New Salt and Drought Stress-Related Factors Involved in Seed Germination. Mol. Biol. Rep..

[47] Mittler R. (2002). Oxidative Stress, Antioxidants and Stress Tolerance. Trends Plant Sci..

[48] Mittler R. (2017). ROS Are Good. Trends Plant Sci..

[49] Sadak M.S., Abd El-Hameid A.R., Zaki F.S.A., Dawood M.G., El-Awadi M.E. (2020). Physiological and Biochemical Responses of Soybean (*Glycine max* L.) to Cysteine Application under Sea Salt Stress. Bull. Natl. Res. Cent..

[50] Ibrahim M.F.M., Faisal A., Shehata S.A. (2016). Calcium Chloride Alleviates Water Stress in Sunflower Plants through Modifying Some Physio-Biochemical Parameters. Am. Eurasian J. Agric. Environ. Sci..

[51] Kaur H., Bhardwaj R.D., Grewal S.K. (2017). Mitigation of Salinity-Induced Oxidative Damage in Wheat (*Triticum aestivum* L.) Seedlings by Exogenous Application of Phenolic Acids. Acta Physiol. Plant..

[52] Prasad T.K., Anderson M.D., Martin B.A., Stewart C.R. (1994). Evidence for Chilling-Induced Oxidative Stress in Maize Seedlings and a Regulatory Role for Hydrogen Peroxide. Plant Cell.

[53] van Breusegem F., Vranová E., Dat J.F., Inzé D. (2001). The Role of Active Oxygen Species in Plant Signal Transduction. Plant Sci..

[54] Neill S.J., Desikan R., Clarke A., Hurst R.D., Hancock J.T. (2002). Hydrogen Peroxide and Nitric Oxide as Signalling Molecules in Plants. J. Exp. Bot..

[55] Vandenabeele S., van der Kelen K., Dat J., Gadjev I., Boonefaes T., Morsa S., Rottiers P., Slooten L., van Montagu M., Zabeau M. (2003). A Comprehensive Analysis of Hydrogen Peroxide-Induced Gene Expression in Tobacco. Proc. Natl. Acad. Sci. USA.

[56] Foreman J., Demidchik V., Bothwell J.H.F., Mylona P., Miedema H., Torres M.A., Linstead P., Costa S., Brownlee C., Jones J.D.G. (2003). Reactive Oxygen Species Produced by NADPH Oxidase Regulate Plant Cell Growth. Nature.

[57] Černý M., Habánová H., Berka M., Luklová M., Brzobohatý B. (2018). Hydrogen Peroxide: Its Role in Plant Biology and Crosstalk with Signalling Networks. Int. J. Mol. Sci..

[58] Wrzaczek M., Brosché M., Kangasjärvi J. (2013). ROS Signaling Loops—Production, Perception, Regulation. Curr. Opin. Plant Biol..

[59] Polidoros A.N., Mylona P.v., Scandalios J.G. (2001). Transgenic Tobacco Plants Expressing the Maize *Cat2* Gene Have Altered Catalase Levels That Affect Plant-Pathogen Interactions and Resistance to Oxidative Stress. Transgenic Res..

[60] Wang H., Yang J., Zhang Y., Hu Y., Wang A., Zhu J., Shen H. (2014). Drought Resistance of Cotton with *Escherichia coli* Catalase Gene *KatE*. Acta Bot. Boreal. Occid. Sin..

[61] Matsumura T., Tabayashi N., Kamagata Y., Souma C., Saruyama H. (2002). Wheat Catalase Expressed in Transgenic Rice Can Improve Tolerance against Low Temperature Stress. Physiol. Plant..

[62] Gondim F.A., Gomes-Filho E., Costa J.H., Alencar N.L.M., Prisco J.T. (2012). Catalase Plays a Key Role in Salt Stress Acclimation Induced by Hydrogen Peroxide Pretreatment in Maize. Plant Physiol. Biochem..

[63] Zhao M.-X., Wen J.-L., Wang L., Wang X.-P., Chen T.-S. (2019). Intracellular Catalase Activity Instead of Glutathione Level Dominates the Resistance of Cells to Reactive Oxygen Species. Cell Stress Chaperones.

[64] Pandey S., Fartyal D., Agarwal A., Shukla T., James D., Kaul T., Negi Y.K., Arora S., Reddy M.K. (2017). Abiotic Stress Tolerance in Plants: Myriad Roles of Ascorbate Peroxidase. Front. Plant Sci..

[65] Ramana Gopavajhula V., Viswanatha Chaitanya K., Akbar Ali Khan P., Shaik J.P., Narasimha Reddy P., Alanazi M. (2013). Modeling and Analysis of Soybean (*Glycine max*. L) Cu/Zn, Mn and Fe Superoxide Dismutases. Genet. Mol. Biol..

[66] Lu W., Duanmu H., Qiao Y., Jin X., Yu Y., Yu L., Chen C. (2020). Genome-Wide Identification and Characterization of the Soybean SOD Family during Alkaline Stress. PeerJ.

[67] Corpas F.J., Fernández-Ocaña A., Carreras A., Valderrama R., Luque F., Esteban F.J., Rodríguez-Serrano M., Chaki M., Pedrajas J.R., Sandalio L.M. (2006). The Expression of Different Superoxide Dismutase Forms Is Cell-Type Dependent in Olive (*Olea europaea* L.) Leaves. Plant Cell Physiol..

[68] Xu J., Yang J., Duan X., Jiang Y., Zhang P. (2014). Increased Expression of Native Cytosolic Cu/Zn Superoxide Dismutase and Ascorbate Peroxidase Improves Tolerance to Oxidative and Chilling Stresses in Cassava (*Manihot esculenta* Crantz). BMC Plant Biol..

[69] Wang Y.C., Qu G.Z., Li H.Y., Wu Y.J., Wang C., Liu G.F., Yang C.P. (2010). Enhanced Salt Tolerance of Transgenic Poplar Plants Expressing a Manganese Superoxide Dismutase from *Tamarix androssowii*. Mol. Biol. Rep..

[70] Yarra R., Wei W. (2021). The NAC-Type Transcription Factor *GmNAC20* Improves Cold, Salinity Tolerance, and Lateral Root Formation in Transgenic Rice Plants. Funct. Integr. Genomics.

[71] Zhang X., Chen L., Shi Q., Ren Z. (2020). *SlMYB102*, an R2R3-Type MYB Gene, Confers Salt Tolerance in Transgenic Tomato. Plant Sci..

[72] Mushke R., Yarra R., Kirti P.B. (2019). Improved Salinity Tolerance and Growth Performance in Transgenic Sunflower Plants via Ectopic Expression of a Wheat Antiporter Gene (*TaNHX2*). Mol. Biol. Rep..

[73] Sahoo D.P., Kumar S., Mishra S., Kobayashi Y., Panda S.K., Sahoo L. (2016). Enhanced Salinity Tolerance in Transgenic Mungbean Overexpressing *Arabidopsis* Antiporter (*NHX1*) Gene. Mol. Breed..

[74] Zhao X., Wei P., Liu Z., Yu B., Shi H. (2017). Soybean Na^+^/H^+^ Antiporter GmsSOS1 Enhances Antioxidant Enzyme Activity and Reduces Na^+^ Accumulation in *Arabidopsis* and Yeast Cells under Salt Stress. Acta Physiol. Plant..

[75] Amini S., Ghobadi C., Yamchi A. (2015). Proline Accumulation and Osmotic Stress: An Overview of *P5CS* Gene in Plants. J. Plant Mol. Breed..

[76] Li Y., Chen Q., Nan H., Li X., Lu S., Zhao X., Liu B., Guo C., Kong F., Cao D. (2017). Overexpression of *GmFDL19* Enhances Tolerance to Drought and Salt Stresses in Soybean. PLoS ONE.

[77] Yang Y., Yu T.F., Ma J., Chen J., Zhou Y.b., Chen M., Ma Y.Z., Wei W.L., Xu Z.S. (2020). The Soybean BZIP Transcription Factor Gene *GmbZIP2* Confers Drought and Salt Resistances in Transgenic Plants. Int. J. Mol. Sci..

[78] Patade V.Y., Bhargava S., Suprasanna P. (2011). Salt and Drought Tolerance of Sugarcane under iso-Osmotic Salt and Water Stress: Growth, Osmolytes Accumulation, and Antioxidant Defense. J. Plant Interact..

[79] Barak S., Farrant J.M. (2016). Extremophyte Adaptations to Salt and Water Deficit Stress. Funct. Plant Biol..

[80] Dar M.I., Naikoo M.I., Rehman F., Naushin F., Khan F.A., Iqbal N., Nazar R., Khan N. (2016). Proline accumulation in plants: Roles in stress tolerance and plant development. Osmolytes and Plants Acclimation to Changing Environment: Emerging Omics Technologies.

[81] Yu Z., Wang X., Zhang L. (2018). Structural and Functional Dynamics of Dehydrins: A Plant Protector Protein under Abiotic Stress. Int. J. Mol. Sci..

[82] Shi H., Ishitani M., Kim C., Zhu J.-K. (2000). The *Arabidopsis thaliana* Salt Tolerance Gene *SOS1* Encodes a Putative Na^+^/H^+^ Antiporter. Proc. Natl. Acad. Sci. USA.

[83] Apse M.P., Sottosanto J.B., Blumwald E. (2003). Vacuolar Cation/H^+^ Exchange, Ion Homeostasis, and Leaf Development Are Altered in a T-DNA Insertional Mutant of *AtNHX1*, the *Arabidopsis* Vacuolar Na^+^/H^+^ Antiporter. Plant J..

[84] Apse M.P., Aharon G.S., Snedden W.A., Blumwald E. (1999). Salt Tolerance Conferred by Overexpression of a Vacuolar Na^+^/H^+^ Antiport in *Arabidopsis*. Science.

[85] Blumwald E. (2000). Sodium Transport and Salt Tolerance in Plants. Curr. Opin. Cell Biol..

[86] Hasegawa P.M., Bressan R.A., Zhu J.-K., Bohnert H.J. (2000). Plant Cellular and Molecular Responses to High Salinity. Annu. Rev. Plant Biol..

[87] Adabnejad H., Kavousi H.R., Hamidi H., Tavassolian I. (2015). Assessment of the Vacuolar Na^+^/H^+^ Antiporter (*NHX1*) Transcriptional Changes in *Leptochloa fusca* L. in Response to Salt and Cadmium Stresses. Mol. Biol. Res. Commun..

[88] Zhang H.-X., Blumwald E. (2001). Transgenic Salt-Tolerant Tomato Plants Accumulate Salt in Foliage but Not in Fruit. Nat. Biotechnol..

[89] Zhao F.-Y., Zhang X.-J., Li P.-H., Zhao Y.-X., Zhang H. (2006). Co-Expression of the *Suaeda salsa SsNHX1* and *Arabidopsis AVP1* Confer Greater Salt Tolerance to Transgenic Rice than the Single *SsNHX1*. Mol. Breed..

[90] Chen H., An R., Tang J.-H., Cui X.-H., Hao F.-S., Chen J., Wang X.-C. (2007). Over-Expression of a Vacuolar Na^+^/H^+^ Antiporter Gene Improves Salt Tolerance in an Upland Rice. Mol. Breed..

[91] Verma D., Singla-Pareek S.L., Rajagopal D., Reddy M.K., Sopory S.K. (2007). Functional Validation of a Novel Isoform of Na^+^/H^+^ Antiporter from *Pennisetum glaucum* for Enhancing Salinity Tolerance in Rice. J. Biosci..

[92] Xue Z.-Y., Zhi D.-Y., Xue G.-P., Zhang H., Zhao Y.-X., Xia G.-M. (2004). Enhanced Salt Tolerance of Transgenic Wheat (*Tritivum aestivum* L.) Expressing a Vacuolar Na^+^/H^+^ Antiporter Gene with Improved Grain Yields in Saline Soils in the Field and a Reduced Level of Leaf Na^+^. Plant Sci..

[93] Fukuda A., Nakamura A., Tagiri A., Tanaka H., Miyao A., Hirochika H., Tanaka Y. (2004). Function, Intracellular Localization and the Importance in Salt Tolerance of a Vacuolar Na^+^/H^+^ Antiporter from Rice. Plant Cell Physiol..

[94] Mishra S., Alavilli H., Lee B., Panda S.K., Sahoo L. (2015). Cloning and Characterization of a Novel Vacuolar Na^+^/H^+^ Antiporter Gene (*VuNHX1*) from Drought Hardy Legume, Cowpea for Salt Tolerance. Plant Cell Tissue Organ Cult..

[95] Qin F., Sakuma Y., Tran L.-S.P., Maruyama K., Kidokoro S., Fujita Y., Fujita M., Umezawa T., Sawano Y., Miyazono K. (2008). *Arabidopsis* DREB2A-Interacting Proteins Function as RING E3 Ligases and Negatively Regulate Plant Drought Stress–Responsive Gene Expression. Plant Cell.

[96] Tizaoui K., Kchouk M.E. (2012). Genetic Approaches for Studying Transgene Inheritance and Genetic Recombination in Three Successive Generations of Transformed Tobacco. Genet. Mol. Biol..

[97] Chuong N.N., Hoang X.L.T., Nghia D.H.T., Nguyen N.C., Thao D.T.T., Tran T.B., Ngoc T.T.M., Thu N.B.A., Nguyen Q.T., Thao N.P. (2021). Ectopic Expression of *GmHP08* Enhances Resistance of Transgenic *Arabidopsis* toward Drought Stress. Plant Cell Rep..

[98] Thu N.B.A., Nguyen Q.T., Hoang X.L.T., Thao N.P., Tran L.S.P. (2014). Evaluation of Drought Tolerance of the Vietnamese Soybean Cultivars Provides Potential Resources for Soybean Production and Genetic Engineering. Biomed Res. Int..

[99] Shu K., Qi Y., Chen F., Meng Y., Luo X., Shuai H., Zhou W., Ding J., Du J., Liu J. (2017). Salt Stress Represses Soybean Seed Germination by Negatively Regulating GA Biosynthesis While Positively Mediating ABA Biosynthesis. Front. Plant Sci..

[100] Wang Y., Jiang L., Chen J., Tao L., An Y., Cai H., Guo C. (2018). Overexpression of the Alfalfa *WRKY11* Gene Enhances Salt Tolerance in Soybean. PLoS ONE.

[101] Wijewardana C., Raja Reddy K., Jason Krutz L., Gao W., Bellaloui N. (2019). Drought Stress Has Transgenerational Effects on Soybean Seed Germination and Seedling Vigor. PLoS ONE.

[102] Patterson B.D., MacRae E.A., Ferguson I.B. (1984). Estimation of Hydrogen Peroxide in Plant Extracts Using Titanium(IV). Anal. Biochem..

[103] Bradford M.M. (1976). A Rapid and Sensitive Method for the Quantitation of Microgram Quantities of Protein Utilizing the Principle of Protein-Dye Binding. Anal. Biochem..

[104] Wang C.J., Yang W., Wang C., Gu C., Niu D.D., Liu H.X., Wang Y.P., Guo J.H. (2012). Induction of Drought Tolerance in Cucumber Plants by a Consortium of Three Plant Growth-Promoting Rhizobacterium Strains. PLoS ONE.

[105] Giannopolitis C.N., Ries S.K. (1977). Superoxide Dismutases: II. Purification and Quantitative Relationship with Water-Soluble Protein in Seedlings. Plant Physiol..

[106] Rodríguez Y., Pérez E., Solórzano E., Meneses A.R., Fernández F. (2001). Peroxidase and Polyphenoloxidase Activities in Tomato Roots Inoculated with *Glomus clarum* or *Glomus fasciculatum*. Cult. Trop..

[107] Le D.T., Aldrich D.L., Valliyodan B., Watanabe Y., Ha C.v., Nishiyama R., Guttikonda S.K., Quach T.N., Gutierrez-Gonzalez J.J., Tran L.-S.P. (2012). Evaluation of Candidate Reference Genes for Normalization of Quantitative RT-PCR in Soybean Tissues under Various Abiotic Stress Conditions. PLoS ONE.

[108] Livak K.J., Schmittgen T.D. (2001). Analysis of Relative Gene Expression Data Using Real-Time Quantitative PCR and the 2^−ΔΔCT^ Method. Methods.

[109] Jiao C., Yang R., Zhou Y., Gu Z. (2016). Nitric Oxide Mediates Isoflavone Accumulation and the Antioxidant System Enhancement in Soybean Sprouts. Food Chem..

[110] Stolf-Moreira R., Medri M.E., Neumaier N., Lemos N.G., Pimenta J.A., Tobita S., Brogin R.L., Marcelino-Guimarães F.C., Oliveira M.C.N., Farias J.R.B. (2010). Soybean Physiology and Gene Expression during Drought. Genet. Mol. Res..

